# Optimization of Toxicity, Biodegradability, and Skin Irritation in Formulations Containing Mixtures of Anionic and Nonionic Surfactants Combined with Silica Nanoparticles

**DOI:** 10.3390/toxics13010043

**Published:** 2025-01-08

**Authors:** Manuela Lechuga, Mercedes Fernández-Serrano, Josefa Núñez-Olea, Juan Francisco Martínez-Gallegos, Francisco Ríos

**Affiliations:** Department of Chemical Engineering, Faculty of Sciences, University of Granada, Campus Fuente Nueva s/n, 18071 Granada, Spain; nlvillen@ugr.es (M.L.); jnolea@ugr.es (J.N.-O.); jfmart@ugr.es (J.F.M.-G.); rios@ugr.es (F.R.)

**Keywords:** toxicity, ecotoxicological risk, biodegradation, skin irritation, fatty-alcohol ethoxylate, ether carboxylic derivative surfactant, silica nanoparticles, mixtures of surfactants

## Abstract

Surfactants play a crucial role in various industrial applications, including detergents and personal care products. However, their widespread use raises concerns due to their potential environmental impact and health risks, particularly in aquatic ecosystems, where they can disrupt the balance of marine life and accumulate in water sources, posing challenges to sustainable development. This study investigates the environmental and health implications of anionic and nonionic surfactants, focusing on their toxicity, biodegradation, and skin irritation potential profiles, especially when combined with silica nanoparticles. Toxicity assessments were conducted using bacteria *Vibrio fischeri* for aquatic toxicity and *Lepidium sativum* seeds for terrestrial plant effects, revealing that individual surfactants like the anionic alkyl ether carboxylic acid EC-R_12–14_E_3_ exhibit high toxicity levels, while the nonionic fatty-alcohol ethoxylate FAE-R_12–14_E_11_ shows comparatively lower environmental impact. The toxicity of surfactant mixtures was analysed, revealing both antagonistic and synergistic effects depending on the surfactants used. The addition of silica nanoparticles generally mitigates the overall toxicity of surfactants, whether used individually or in mixtures. Biodegradation studies followed OECD 301E and 301F guidelines, indicating that individual surfactants generally meet or approach the mineralization threshold, whereas the addition of nanoparticles reduced biodegradation efficacy. Potential skin irritation was predicted through the zein number (ZN), finding that some surfactant combinations with silica nanoparticles reduce irritation levels, highlighting their potential for safer formulation in products that come into direct contact with the skin. Overall, the findings emphasize the need for careful selection of surfactant mixtures and nanoparticle integration to minimize environmental toxicity and potential skin irritation and increase their biodegradability.

## 1. Introduction

Surfactants are amphiphilic molecules consisting of a lipid-soluble segment and a water-soluble segment, allowing them to reduce surface tension and interact with both lipophilic and hydrophilic substances. These properties make surfactants indispensable in various industries such as cosmetics, pharmaceuticals, detergents, disinfectants, and personal care products [1,2]. In 2022, CESIO reported a production of over 2.8 million tonnes of surfactants in Europe, highlighting their widespread use and economic significance. Surfactants play a main role in formulas such as laundry and dishwasher detergents, all-purpose cleaners, cosmetic and personal care products, shampoos and conditioners, bath gel and liquid soaps, creams and lotions, emulsifiers in foods, herbicides and pesticides, drug formulations, and paints and coatings [3].

While surfactants are essential in various industries due to their versatile properties, their environmental impact [4], potential for skin irritation [5], and toxicity to aquatic organisms [6] highlight the need for continuous evaluation and regulation. The development of safer and more sustainable alternatives, coupled with stringent biodegradability testing, will be critical in reducing the ecological footprint of surfactants and ensuring their responsible use.

The European Regulation (EC) No 648/2004 on detergents [7] mandates strict rules on the biodegradability of surfactants to ensure environmental protection. This regulation defines detergents as products containing surfactants for cleaning purposes and standardizes their labelling and restrictions on biodegradability. This framework promotes safer use and environmental monitoring of these compounds. The biodegradability of surfactants is closely linked to their chemical structure [8]. Beyond biodegradability, surfactant toxicity plays a key role in their environmental impact. Elevated concentrations of surfactants in water bodies can disrupt biological membranes and essential cellular processes in aquatic organisms, leading to adverse effects on biodiversity [9,10]. Furthermore, incomplete biodegradation of some surfactants can result in their accumulation in sediments and groundwater, posing long-term risks to aquatic ecosystems [11]. To mitigate environmental and health concerns, there is an increasing focus on developing eco-friendly surfactants that offer improved biodegradability and reduced toxicity [1]. Regulatory frameworks, including the REACH Regulation [12], require rigorous testing, such as the OECD 301F biodegradation test [13], to ensure compliance with environmental standards and encourage the adoption of more sustainable alternatives.

Regarding the human health implications of surfactants, and despite their widespread use in daily-use products, surfactants can cause skin irritation. This is due to their ability to disrupt the skin’s lipid barrier, leading to dryness, inflammation, and conditions such as contact dermatitis [14,15]. The potential for irritation underscores the importance of conducting thorough toxicity assessments, especially in cosmetic and personal care products that come into direct contact with the skin.

Among commercial surfactants, fatty-alcohol ethoxylates are the most economically significant group of nonionic surfactants, extensively used in both domestic and commercial detergents, household cleaners, and personal care products [3]. Their versatility, high performance, and cost-effectiveness make them a popular choice in various cosmetic and personal care products, including skincare items, shampoos, and body creams [3]. Ether carboxylic acid derivative surfactants are anionic surfactants that enhance the foaming properties of detergents and reduce irritation levels. This makes them ideal co-surfactants in detergents and skin-contact formulations.

Mixtures of surfactants with varying properties (anionic, nonionic, and amphoteric) are frequently used in formulations due to their superior performance compared to single surfactants. These combinations are particularly effective in enhancing efficiency and stability while also reducing toxicity, costs, and the potential for skin and eye irritation [16,17]. As a result, once these products are used, surfactant mixtures of different types are typically discharged into water streams together. As many of these formulations are applied directly to the skin, such as personal care products, ensuring that surfactant mixtures are mild and safe is critical for reducing potential adverse reactions.

Silica nanoparticles are increasingly used in detergents and cosmetics due to their unique properties. In detergents, they act as anti-redeposition agents and mild abrasives, enhancing cleaning efficiency without damaging surfaces [18,19]. In cosmetics, they are used as texturizing agents, oil absorbents, and stabilizers in emulsions, improving the feel of the products and pigment dispersion [20]. It is especially useful for transforming cosmetic oils into highly viscous gels, creating transparent products. Additionally, these nanoparticles aid in the even distribution of pigments in colour cosmetics and prevent the clumping of active ingredients, ensuring better formulation stability. Due to their widespread use in recent years, numerous authors have considered the release and impact of nanoparticles in the environment and wastewater [21,22].

The environmental impact of common surfactants, particularly their aquatic toxicity, has been extensively studied in various aquatic organisms [23,24,25,26]. However, there is a significant gap in research that examines the combined effects of surfactants or evaluates the potential for synergistic, additive, or antagonistic interactions. While individual surfactant studies provide valuable insights, they often fail to capture real-world scenarios where multiple surfactants are present simultaneously. As with their physicochemical properties, the combination of surfactants can lead to synergistic or antagonistic effects on their environmental impact, toxicity, and skin irritation. Similarly, to our knowledge, the environmental impact of mixtures of surfactants and silica nanoparticles, as well as their interactions, has been scarcely studied. To fill this knowledge gap, it is crucial to conduct holistic research that investigates the effects of multiple surfactants simultaneously, in the presence or absence of silica nanoparticles, with a focus on elucidating the interactions within the mixtures. This approach will help to better understand their environmental impact, toxicity, and skin irritation, and provide a more accurate representation of their behaviour in real-world applications.

In this study, the toxicity, biodegradation, and skin irritation of three anionic surfactants (ether carboxylic derivative) and a nonionic surfactant (fatty-alcohol ethoxylate) were evaluated, as individual surfactants and as mixtures, including the effect of the addition of silica nanoparticles.

## 2. Materials and Methods

### 2.1. Surfactants and Nanoparticles

In this work, four commercial surfactants were studied, three ether carboxylic acid derivatives with different alkyl chains and degrees of ethoxylation, (Kao Corporation. Emmerich, Germany) and a fatty-alcohol ethoxylate (Kao Corporation, Germany).
**R-O(CH_2_-CH_2_O)_n_-CH_2_-COO-X****R(-O-CH_2_-CH_2_)_n_-OH**Ether carboxylic derivative surfactants  (EC-R_12–14_E_10_, EC-R_12–14_E_3_, EC-R_8_E_5_)Fatty-alcohol ethoxylates  (FAE-R_12–14_E_11_)

Table 1 shows the abbreviation, the International Nomenclature of Cosmetics Ingredients (INCI name), the length of the alkyl chain (R), the ethoxylation degree (E),% active matter, hydrophilic–lipophilic balance (HLB) and critical micelle concentration (CMC) of the surfactants tested. Hydrophilic fumed silica nanoparticles (Aerosil^®^ 200, Evonik Industries. Essen, Germany) were integrated into the surfactant formulation to enhance its properties. Table 1 outlines key characteristics provided by the supplier, including the mean diameter (D_m_), surface area (S), and tamped density (d). Aerosil^®^ 200 is frequently employed in cosmetic formulations as a thickening and stabilizing agent.

### 2.2. Acute Toxicity Tests

#### 2.2.1. Luminescence Inhibition Assay in *Vibrio fischeri*

The toxicity measurement is based on the luminous intensity of marine bacteria of the strain *V. fischeri* NRRLB11177 after exposure to a toxic substance. Measurements were taken using the LumiStox 300 system, which includes a bioluminescence measuring instrument and an incubation unit, in accordance with the ISO 11348-2:2007 guideline [27]. The luminescent bacteria, which were dehydrated and frozen at −18 °C, were reactivated with a suspension provided by Dr. Lange. The assay conditions were maintained at a pH of 7.0 with a NaCl concentration of 2%. Triplicate measurements were taken at incubation times of 30 min. The toxicity values were determined as EC_50_, representing the surfactant concentrations that inhibit 50% of the bioluminescence after 30 min of exposure. If necessary, the sample was filtered prior to the assay.

#### 2.2.2. Germination Test on *Lepidus sativum*

The germination test on *L. sativum* (cress) seeds is a static test of acute toxicity. This test allows for evaluating the phytotoxic effects produced in the seed germination process and the development of seedlings during the first days of growth (72 h). The garden cress test with *L. sativum* was carried out according to ISO-18763:2016 [28]. The inhibition in the elongation of the root and stem was determined.

Briefly, 25 *L. sativum* seeds were placed in a well-distributed manner in Petri dishes (100 mm Ø) containing filter paper saturated with 5 mL of the test solution. Distilled water was used as a control, and zinc (II) was used as a toxic reference compound. Each Petri dish was covered and sealed to prevent moisture loss. For 72 h, at 25 °C, and in the absence of light, the different samples were incubated in triplicate. Once the incubation was completed, the length of the root and stem of each germinated seed was measured with the help of graph paper. The inhibition of the root and stem growth by the test solutions was assessed in comparison with the control.

### 2.3. Biodegradation Tests

#### 2.3.1. Static Biodegradation Test

The static biodegradation tests were conducted according to the OECD 301E guidelines [13], which focus on the removal of organic compounds measured as dissolved organic carbon (DOC). Solutions of the surfactant and surfactant–co-surfactant mixtures, serving as the sole carbon source for microorganisms, were tested in a mineral medium inoculated and incubated under aerobic conditions in the dark for 28 days. The initial concentration of the test substance for surfactant and surfactant–co-surfactant mixtures was set at 25 mg·L^−1^.

The surfactant solution, for which biodegradability was to be determined, was inoculated with 0.5 mL of water from the secondary treatment stage of a sewage treatment plant (STP) operating with activated sludge (plus code: 597G+W2, Granada, Spain). The biodegradation process was monitored by measuring the residual surfactant concentration over time through DOC determinations in samples, filtered through a 0.45 µm Millipore membrane, using the total organic carbon (TOC) analyser Shimadzu VCSH/CSH (Shimadzu Co., Kyoto, Japan). Reference assays were performed with an easily biodegradable surfactant (linear alkylbenzene sulfonate) to determine the activity of the microbial population present in the test medium. Abiotic assays were conducted in the presence of HgCl_2_ to confirm this, revealing that the residual surfactant levels remained around 100% throughout the biodegradation period. All samples were tested in duplicate. The experimental setup included two flasks for the blank, two for the reference surfactant, two for the abiotic assay, and two for each of the surfactant concentrations tested.

#### 2.3.2. Manometric Respirometry Test

The manometric respirometry test, using the OECD 301F method [13], was applied to surfactant–nanoparticle mixtures to assess their biodegradation progress and determine their final mineralization. A substance or mixture of substances was considered “readily biodegradable” if at least 60% of the theoretical oxygen demand (ThOD) or 60% of the dissolved organic carbon (DOC) was mineralized to CO_2_ within 28 days. Biodegradation was quantified as a percentage of the oxygen consumed by the microbial population (biological oxygen demand (BOD)) to degrade the test substance, relative to the initial ThOD, adjusted for uptake observed in a parallel blank with only the inoculum (endogenous respiration) [29].

The test was performed using a known concentration of surfactant or mixtures (50 mg ThOD·L^−1^) as the sole organic carbon source in a mineral medium inoculated with pre-treated secondary effluent from the local sewage treatment plant (STP) operating with activated sludge (plus code: 597G+W2, Granada, Spain). The preparation of the mineral medium involved the addition of 1 L of water; 10 mL of solution A; and 1 mL each of solutions B, C, and D, with pH adjusted to 7.4 using 1M NaOH or 1N HCl. The solutions were as follows: Solution A: 8.5 g KH_2_PO_4_, 21.75 g K_2_HPO_4_, 33.4 g Na_2_HPO_4_•2H_2_O, and 0.5 g NH_4_Cl in 1 L; Solution B: 36.40 g CaCl_2_•2H_2_O in 1 L; Solution C: 22.5 g MgSO_4_•H_2_O in 1 L; and Solution D: 0.25 g FeCl_3_ in 1 L.

For surfactant and nanoparticle mixtures, the medium contained 50 mg ThODL^−1^ of surfactants and 250 mg·L^−1^ of nanoparticles. In the blanks, no carbon-based substance was added. The aerated inoculum was added to achieve a microorganism concentration of 30 mg of total solid suspend (TSS) per litre, and the pH was adjusted to 7.4 ± 0.2. Reactors were then sealed and maintained in darkness, under constant agitation, at 22 ± 1 °C for 28 days. The Oxitop^®^ system recorded BOD measurements daily. Samples were tested in triplicate.

The elemental composition of the surfactants was used to calculate their theoretical oxygen demand (ThOD) based on Equation (1) (OECD 301, 1992):

(1) $$ThOD(\frac{mgO_{2}}{mgSubstance})=\frac{16\cdot \left[2\cdot C+\frac{1}{2}\cdot \left(H-Cl\right)+\frac{5}{2}\cdot N+3S+\frac{5}{2}\cdot P+\frac{1}{2}\cdot Na-O\right]}{MW}$$

In this equation, MW represents the molecular weight of the surfactant, while C, H, Cl, N, Na, O, P, and S denote the number of atoms of carbon, hydrogen, chlorine, nitrogen, sodium, oxygen, phosphorus, and sulphur per molecule, respectively.

### 2.4. Irritation Potential Using the Zein Method

The irritant potential of surfactants, their mixtures, and the addition of silica nanoparticles was assessed using a modified zein method [17,30,31]. This method is used to assess the potential irritation of chemicals or cosmetics on the skin. It is based on the interaction of a substance with proteins, particularly with the protein zein, which is a protein derived from corn. The modified zein method is recognized as an in vitro alternative to predict dermal irritation without the need to perform tests on animals. This method involves three stages to determine the amount of protein (zein) solubilized in a surfactant-containing solution: (1) zein denaturation, (2) oxidation and mineralization of organic matter, and (3) nitrate content measurement. All experiments were conducted at room temperature and performed in triplicate. For these tests, the final concentration of each component, whether pure or in a mixture, was set at 0.5% and 1% wt. These concentrations reflect typical dosages found in cosmetic, detergent, and personal hygiene products that come into direct contact with the skin. The zein number (ZN), expressed as mg of nitrogen (N) released per 100 g of surfactant solution, was calculated using the following formula:

ZN = c/10(2)

where c is the nitrogen concentration measured spectrophotometrically in ppm (mg·1000 mL^−1^). Given that the density of the testing solution is 1 g·mL^−1^, c is ultimately expressed as mg·1000 g^−1^. Two control cases were used for validation: a negative control with deionized water and a positive control with 1% wt. sodium lauryl sulphate (SLS), a well-known surfactant with high irritant potential.

## 3. Results and Discussion

### 3.1. Toxicity

#### 3.1.1. Toxicity for Individual Surfactants

The toxicity of individual anionic and nonionic surfactants on *V. fischeri* and *L. sativum* was evaluated, and their EC_50_ results are presented in Table 2. For *V. fischeri*, toxicity values are expressed as EC_50_ values, determined using a logarithmic plot of sample concentration versus the percentage reduction in light intensity after a 30-min exposure period. This indicates the concentration at which there is a 50% reduction in bioluminescence. Similarly, for *L. sativum*, toxicity values are also expressed as EC_50_ values, which represent the concentration at which 50% growth inhibition is observed. This was measured by assessing the root and stem elongation after a 72-hour exposure period. The specific growth parameters, such as root length reduction, were used to determine the EC_50_ values.

The toxicity results for the four surfactants revealed significant differences in their toxic effects. In the case of individual surfactants, values ranged from 3.58 mg·L^−1^ to 32.76 mg·L^−1^ for *V. fischeri* and from 32.02 mg·L^−1^ to 338.17 mg·L^−1^ and 60.11 mg·L^−1^ to 97.15 mg·L^−1^ in the case of *L. sativum* root and stem growth, respectively.

The results revealed a higher sensitivity to the surfactants tested for bacteria *V. fischeri* than to *L. sativum*. Similar results were found in previous studies, in which bacteria *V. fischeri* showed the highest sensitivity to surfactants compared to other organisms of higher level such as microcrustaceans *D. magna* or freshwater microalgae [9]. Bacteria can be more sensitive to exposure to surfactants due to several factors. They often lack specialized mechanisms to defend against certain toxic substances, exhibit high susceptibility to compounds that target cellular membranes or essential enzymes, and experience greater relative exposure due to their small size. In contrast, higher organisms, such as plants, possess more advanced enzymatic systems capable of metabolizing certain toxic compounds, which makes them less sensitive to toxins like surfactants. For all the surfactants tested with *V. fischeri*, the EC_50_ values were lower than the CMC of the surfactant. In contrast, for *L. sativum*, the opposite trend was observed in the case of FAE-R_12–14_E_11_ and EC-R_12–14_E_3_, for which the EC_50_ values exceeded the CMC. Since the toxic effects of surfactants are often linked to the presence of free monomers that interact with the biological systems of organisms, this observation may explain the higher sensitivity of bacteria to surfactant toxicity compared to plants.

Furthermore, a clear relationship was identified between EC_50_ values for *V. fischeri*, the hydrophilic–lipophilic balance (HLB), and the CMC for the three anionic surfactants tested. The relationships observed and their corresponding coefficients of determination were as follows:

EC_50_, *V. fischeri* mg·L^−1^ = 0.445·CMC − 13.147 (R^2^ = 0.9406) (3)

EC_50_, *V. fischeri* mg·L^−1^ = 3.8613·HLB − 19.199 (R^2^ = 0.9624) (4)

The toxicity of the surfactants on the root and stem of *L. sativum* presented a different pattern compared to *V. fischeri*. FAE-R_12–14_E_11_ demonstrated the least toxicity to roots and stems among all the surfactants tested, suggesting that this nonionic surfactant might be safer for terrestrial plants in comparison with the anionic surfactants assayed. However, its effect on the stem (EC_50_ = 97.15 mg·L^−1^) was more pronounced compared to the root (EC_50_ = 338.17 mg·L^−1^), which could influence plant growth depending on the part of the plant considered.

In contrast, EC-R_12–14_E_10_ showed the highest toxicity to the roots (EC_50_ = 32.02 mg·L^−1^) and stems (EC_50_ = 60.11 mg·L^−1^) of *L. sativum*, indicating that this anionic surfactant could be particularly harmful to plant growth at lower concentrations. This aligned with its relatively high toxicity in both *V. fischeri* and *L. sativum*. EC-R_8_E_5_ displayed medium toxicity levels for both root and stem, with values of 53.61 mg·L^−1^ and 64.77 mg·L^−1^, respectively. While it showed lower root toxicity compared to EC-R_12–14_E_3_ and EC-R_12–14_E_10_, its impact should not be overlooked, especially at high concentrations.

For *L. sativum* (stem), a clear relationship was observed between EC_50_ values, the hydrophilic–lipophilic balance (HLB), and the critical micelle concentration (CMC) for the three anionic surfactants tested. The identified relationships and their respective coefficients of determination were as follows:

EC_50_, *L. sativum* mg·L^−1^ = 1.0453·CMC − 28.894 (R^2^ = 0.8697) (5)

EC_50_, *L. sativum* mg·L^−1^ = 8.787·HLB − 40.388 (R^2^ = 0.8333) (6)

#### 3.1.2. Toxicity of Surfactant Mixtures

Table 3 presents the toxicity values of the different mixtures tested. Three types of mixtures were tested: binary mixtures (1:1) of the nonionic surfactant with each anionic surfactant, binary mixtures of the individual surfactants combined with the silica nanoparticles A200, and ternary mixtures (1:1) of nonionic–anionic surfactants with silica nanoparticles. Toxicity tests were conducted using *V. fischeri* and *L. sativum* as biological models. The results indicated significant correlations between the toxicity outcomes of both test organisms, suggesting consistent trends in the toxicological behaviour of the mixtures across both bioassays.

As with the toxicity of individual surfactants, the binary mixtures of the surfactants had greater toxic effects on *V. fischeri* bacteria than on *L. sativum*, with EC_50_ values ranging from 10.78 to 20.95 mg·L^−1^ for *V. fischeri* and from 39.48 to 116.41mg·L^−1^ for *L. sativum*. The model of toxic units (MTU) [32,33,34,35] was applied to quantify the interactions between toxicants in binary surfactant mixtures: FAE-R_12–14_E_11_ with EC-R_12–14_E_3_, FAE-R_12–14_E_11_ with EC-R_12–14_E_10_, and FAE-R_12–14_E_11_ with EC-R_8_E_5_. This approach helps predict whether the principles of concentration or response addition apply. The toxic unit for each component in the mixture (TU_i_) was calculated as the ratio between the concentration of the toxicant in the mixture [i] and its half-maximal effective concentration (EC_50i_) (Equation (7)). The total toxic unit of the mixture (TU_mix_) is the sum of the TU_i_ values of the individual components (Equation (7)).

(7) $${TU}_{i}=\frac{\left[i\right]}{{EC}_{50i}}$$

(8) $${TU}_{mix}={TU}_{A}+{TU}_{B}=\frac{\left[A\right]}{{EC}_{50A}}+\frac{\left[B\right]}{{EC}_{50B}}$$

According to existing research and previous studies [36], a simple additive effect (concentration addition) is observed when 0.8 < TU_mix_ < 1.2, indicating that both surfactants act with the same mode of action (MoA). A value of TUmix ≤ 0.8 implies synergism (more than the additive effect), that is, the mixture is more toxic than the individual surfactants, while TU ≥ 1.2 suggests antagonism (less than the additive effect), that is, the mixture is less toxic than the surfactants individually [32]. If the toxicants act independently (response addition), the predicted toxic unit (TU_r_) is calculated using Equations (9) and (10). If TU_r_ ≈ TU_mix_, response addition can be expected.

(9) $$If\;{TU}_{A}>{TU}_{B}\;\;{TU}_{r}=1+\frac{{TU}_{B}}{{TU}_{A}}$$

(10) $$If\;{TU}_{A}<{TU}_{B}\;\;{TU}_{r}=1+\frac{{TU}_{A}}{{TU}_{B}}$$

Values of TU_A_, TU_B_, TU_mix_, and TU_r_, along with conclusions regarding the mode of action for the mixtures tested on *V. fischeri* and *L. sativum*, are summarized in Table 4. The results application of the MTU to the binary surfactant mixtures revealed three varied types of toxicological interactions: antagonistic (less than additive), synergistic (more than additive), and concentration addition.

In the case of toxicity tests with bacteria *V. fischeri*, the mixture FAE-R_12–14_E_11_-EC-R_12–14_E_10_ exhibited an antagonistic effect, with a TU_mix_ of 1.53, indicating a less than additive interaction. The mixture FAE-R_12–14_E_11_-EC-R_12–14_E_3_ also demonstrated an interesting antagonistic effect (less than additive) effect, with a TU_mix_ of 1.91, meaning that the combined EC_50_ exceeded the expected value based on the individual components, and therefore the binary mixture was less toxic to bacteria *V. fischeri*. Finally, the toxicity of the mixture FAE-R_12–14_E_11_-EC-R_8_E_5_ followed concentration addition principles (TU_mix_ = 1.09, TU_r_ = 3.47), indicating that the surfactants behaved additively, without significant interaction effects. According to the results, it can be observed that for the mixture with ether carboxylic acid derivative with the shortest alkyl chain (EC-R_8_E_5_) and the highest CMC and HLB, its type of action differed from that of the other two surfactants of the same family, highlighting the influence of the surfactant structure on the toxic effects of the mixtures.

For *L. sativum* (root), the interaction between FAE-R_12–14_E_11_ and EC-R_12–14_E_10_ showed antagonism (TU_mix_ = 1.99. However, for *L. sativum* (stem), this same mixture exhibited an additive effect (TU_mix_ = 0.90), suggesting organ-specific responses to the surfactant combinations. The mixture of FAE-R_12–14_E_11_ and EC-R_12–14_E_3_ also showed less than additive behaviour for roots (TU_mix_ = 1.35), although not as clear as in the previous case, and close to response addition (TU_r_ = 1.10), while in stems, it showed a more than additive effect (TU_mix_ = 0.66). This indicates differential toxicity mechanisms in the plant depending on the tissue. The combination of FAE-R_12–14_E_11_ and EC-R_8_E_5_ consistently demonstrated a more than additive effect for both root and stem (TU_mix_ = 0.43 for roots and TU_mix_ = 0.68 for stems), meaning that this mixture exerted a synergistic effect on the toxicity to *L. sativum* and may pose a higher environmental risk, especially for plant tissues, due to its synergistic interaction. Again, the mixture involving the surfactant with the shorter chain length and higher CMC and HLB led to a different type of action.

Table 3 also shows the toxicity results of mixtures of surfactants and silica nanoparticles. The individual toxicity of silica nanoparticles to bacteria *V. fischeri* was previously analysed [21], showing that they can be considered non-toxic, since the percentage of inhibition barely exceeds 10%. The results indicate that A200 nanoparticles generally reduce the overall toxicity of the surfactants, mixed or not, as evidenced by the higher EC_50_ values in most cases when A200 is present in comparison with its absence. The case of the FAE-R_12–14_E_11_ and A200 mixture for *L. sativum* (root) is particularly striking, as the EC_50_ value was up to 2.3 times higher than that of the individual surfactant. This could be related to the adsorption phenomena of surfactants onto the surface of silica nanoparticles, which have been previously reported for FAE-R_12–14_E_11_ and EC-R_12–14_E_3_ [21,37], indicating that they may reduce the capacity of the surfactants to alter the cell membranes, thus reducing their toxicity.

In the case of ternary mixtures composed of the FAE-R_12–14_E_11_ surfactant, each of the three ether carboxylic acid derivatives, and A200 silica nanoparticles, the effect regarding A200 incorporation was very similar. Generally, the EC_50_ values obtained were higher than those of binary mixtures without A200. Therefore, it can be confirmed that the incorporation of silica nanoparticles also helps reduce the toxicity of surfactant mixtures, making them a good alternative for inclusion in formulations from an environmental perspective.

### 3.2. Biodegradation

#### 3.2.1. Static Biodegradation Test

**Figure 1:**
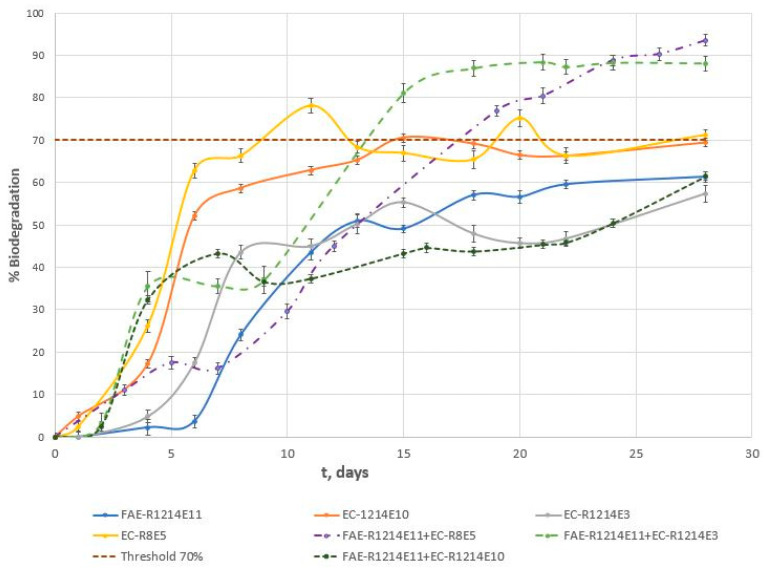
Evolution of ultimate biodegradation over time of single surfactants and binary mixtures (1:1 *w*/*w*).

The biodegradation of surfactants and their binary mixtures was assessed under aerobic conditions following the OECD 301E guidelines for ready biodegradability [13]. The process was tracked by measuring the dissolved organic carbon (DOC) in the samples over time. While surfactant sorption can significantly affect environmental outcomes, it was deemed negligible in these tests due to minimal biomass formation. Abiotic control tests, using HgCl_2_ to inhibit biological activity, confirmed that surfactant concentrations remained at nearly 100% throughout the degradation period. This suggests that abiotic factors did not contribute to surfactant breakdown, confirming the reliability of the biodegradation data collected. Figure 1 illustrates the progression of ultimate biodegradation of the surfactants and binary mixtures over the testing period. The assays were initiated with surfactant concentrations of 25 mg·L^−1^.

To compare and quantify the biodegradation test, two key parameters of the biodegradation profiles [22] were defined and analysed: (a) Half-life (t_1/2_) is the time required for the substrate concentration to decrease by 50% from the start of the biodegradation process. This value is determined using graphical methods based on the biodegradation curve. (b) Surfactant biodegradability (B) is defined as the percentage of surfactant that is biodegraded after 50 h of assay. Half-time and surfactant biodegradability results are shown in Table 5, together with mineralization values obtained using Equation (11).

(11) $$Mineralization\;(\%)=\frac{{\left[DOC\right]}_{i}-{\;[DOC]}_{f}}{{\;[DOC]}_{i}}\cdot 100$$

where

- DOC_i_ is the dissolved organic carbon at the beginning of the test;
- DOC_f_ is the dissolved organic carbon at the end of the test.

The biodegradation data revealed significant differences in the mineralization rates among the surfactants and their mixtures. EC-R_8_E_5_ demonstrated the highest level of biodegradability, achieving 71.20% mineralization with a relatively low t_1/2_ of 118.2 h, indicating rapid degradation under experimental conditions. This anionic surfactant was the only one to surpass the 70% threshold set by the OECD 301 E guidelines, qualifying it as readily biodegradable. In contrast, the anionic surfactant EC-R_12–14_E_3_ exhibited the lowest biodegradation performance, with a mineralization level of 57.35% and the highest t_1/2_ of 330.9 h, indicating the slowest degradation rate. For surfactant mixtures, the results were similarly varied. A comparison of the anionic surfactants indicated that the most biodegradable was the surfactant with the lowest alkyl chain (EC-R_8_E_5_). For surfactants with the same alkyl chain length (FAE-R_12–14_E_11,_ EC-R_12–14_E_10_), the surfactant with the lowest degree of ethoxylation reached higher biodegradability.

Mixtures such as FAE-R_12–14_E_11_ with EC-R_12–14_E_10_ displayed slower biodegradation, with a t_1/2_ of 564.12 h, suggesting a significant delay in degradation compared to the individual surfactants. However, the combination of FAE-R_12–14_E_11_ with EC-R_8_E_5_, which present the lowest and highest biodegradation, respectively, showed exceptional performance, achieving 93.49% mineralization with a moderate t_1/2_ of 310.2 h, surpassing the readily biodegradable threshold and indicating synergistic effects that enhance mineralization.

#### 3.2.2. Manometric Biodegradation Test

**Figure 2:**
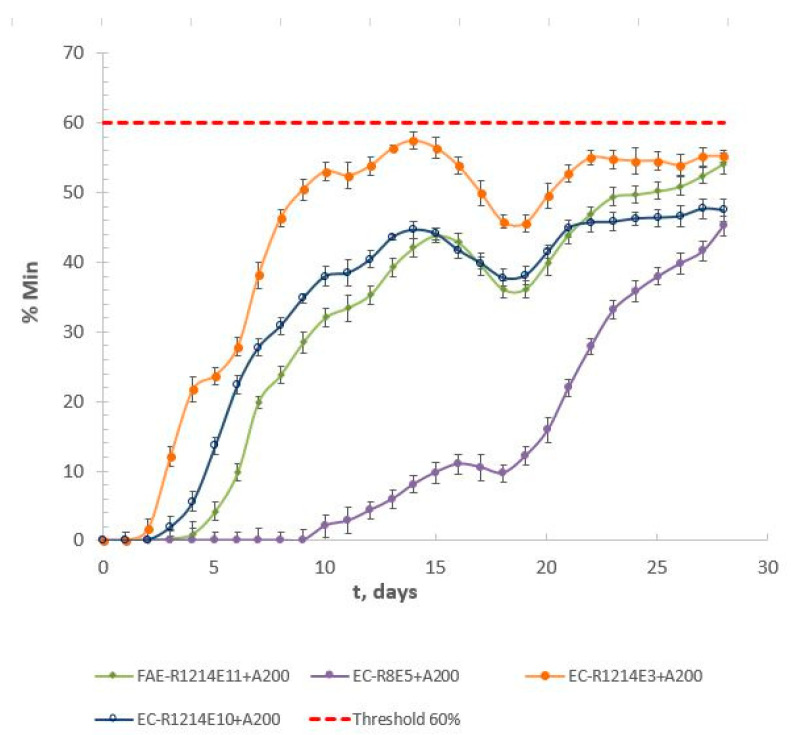
Evolution of mineralization over time using mixtures of surfactants (50 mgThOD·L^−1^) and nanoparticles (250 mg·L^−1^).

The aerobic biodegradation of individual surfactants with nanoparticles was evaluated using the OECD 301F method over a 28-day period [13]. Following the OECD 301F guidelines, the initial concentration of the test substance was set at 50 mg ThODL^−1^ to ensure an adequate amount of biodegradable material that would stimulate microbial activity without overwhelming the system. This approach avoids the introduction of excessive particulate matter that could interfere with accurate CO_2_ measurements. In addition, the nanoparticles were introduced at an initial concentration of 250 mg·L^−1^. To assess the biodegradation process and quantify the final mineralization, we calculated the final mineralization percentage (%Min) according to Equation (12), which represents the total biodegradation achieved after the 28-day test period. Figure 2 illustrates the evolution of mineralization over time using mixtures of surfactants and nanoparticles.

(12) $$Biodegradability\;(\%)=\frac{{\left[BOD\right]}_{t}-{\;[BOD]}_{bt}}{ThOD}\cdot 100$$

where

- BOD_t_ is the biological oxygen demand measured using the Oxitop-C head;
- BOD_bt_ is the biological oxygen demand of the blank;
- ThOD is the theoretical total oxygen required to transform all of test substances into CO_2_ and water.

The biodegradation results (Table 5 and Table 6) showed significant differences in mineralization between individual surfactants and their combinations with silica nanoparticles (A200). When surfactants were combined with silica nanoparticles, a general decrease in mineralization percentages was observed for all the tested surfactants, and none of the surfactant–nanoparticle combinations reached the minimum 60% mineralization level required to be considered “readily biodegradable” according to the OECD 301F guidelines [13]. For instance, the combination of EC-R_8_E_5_ with A200 showed a significant reduction in biodegradation, reaching only 45.25% mineralization. This pattern suggests that the presence of nanoparticles might be interfering with the biodegradation process, potentially due to surfactant adsorption on the nanoparticle surface or the formation of surfactant/NP complexes that affect surfactant availability to the microorganisms responsible for biodegradation [38,39]. Comparatively, FAE-R_12–14_E_11_ exhibited a smaller decrease in biodegradability when combined with A200 (54.18%), indicating that while nanoparticles affected its biodegradation, the impact was less pronounced than for other surfactants, like EC-R_12–14_E_10_, which decreased to 47.50%. This may suggest that the interaction between nanoparticles and surfactants depends on the specific characteristics of each surfactant, such as molecular structure or affinity with the nanoparticles.

### 3.3. Potential Skin Irritation

The zein number (ZN) (Section 2.3) provides an indication of surfactant-induced potential skin irritation. According to the scale established by [31], surfactants can be classified as non-irritant, moderately irritant, irritant, or strongly irritant. Table 7 presents the ZN values obtained for all the tested surfactants and mixtures. The final concentration of each component, whether individual or in a mixture, was set at 0.5% and 1% wt. Notably, 92.8% of the surfactants and mixtures tested at the 0.5% concentration were classified as non-irritating, indicating minimal irritation potential. The concentration of the surfactants had a notable influence on irritation values. Across all the surfactants and their mixtures, ZN values consistently increased at the 1% concentration compared to 0.5%, highlighting a dose-dependent response in irritation potential. For example, FAE-R_12–14_E_11_ had a ZN of 48.91 at 0.5% and 55.6 at 1%, while EC-R_12–14_E_10_ showed an increase from 154.69 at 0.5% to 201.1 at 1%.

Anionic surfactants showed a higher potential for irritation compared to nonionic surfactants, primarily due to their greater ability to solubilize zein protein [40,41,42]. ZN values support this observation, establishing a decreasing order of irritation potential for the tested surfactants as follows: EC-R_12–14_E_10_ > EC-R_12–14_E_3_ > EC-R_8_E_5_ > FAE-R_12–14_E_11_. The addition of nonionic surfactants to mixtures containing anionic surfactants significantly moderated potential irritation, as evidenced by the reduction in ZN values. Nonionic surfactants, such as FAE-R_12–14_E_11_, mitigated the irritant effects of anionic surfactants like EC-R_12–14_E_10_, EC-R_12–14_E_3_, and EC-R_8_E_5_. This moderation can be attributed to a reduction in charge density and decreased protein solubilization, key factors associated with irritation. The increase in ZN from 0.5% to 1% was more pronounced in individual anionic surfactants (EC-R_12–14_E_10_, EC-R_12–14_E_3_, and EC-R_8_E_5_), reinforcing the idea that anionic surfactants had a higher irritation potential, while combinations with FAE-R_12–14_E_11_ appeared to moderate this effect.

The results indicate that adding silica nanoparticles (A200) generally increases the ZN values slightly, implying a mild enhancement in irritation potential, especially at 1% concentration. However, ternary formulations combining fatty-alcohol ethoxylate with any of the tested ether carboxylic surfactants in the presence of silica nanoparticles exhibited a notable reduction in irritation compared to formulations without nanoparticles or binary surfactant combinations. Notably, the FAE-R_12–14_E_11_/EC-R_12–14_E_3_/A200 mixture showed the lowest ZN values, even at 1% concentration (8.0 ± 1.60), highlighting its potential as a low-irritation formulation for sensitive applications. A possible explanation, based on findings from other authors [17,43,44], is that the formation of core–shell structures in these combinations, together with the reduction in charge density due to the nonionic surfactant, contributes to decreased protein solubilization, thereby mitigating dermal irritation.

## 4. Conclusions

The conclusions drawn from this study provide significant insights into the environmental and potential skin irritation of using anionic and nonionic surfactants, particularly when integrated with silica nanoparticles (A200). Firstly, toxicity assessments revealed pronounced differences between the individual surfactants tested and their formulated products. EC-R_12–14_E_3_ exhibited the highest toxicity levels to both aquatic and plant test organisms, while FAE-R_12–14_E_11_ demonstrated the lowest toxicity, positioning it as a relatively more environmentally benign alternative.

The interactions between different surfactant mixtures were found to vary based on specific combinations and the test organism, exhibiting both synergistic and antagonistic effects. For instance, the combination of FAE-R_12–14_E_11_ and EC-R_12–14_E_10_ displayed complex interactions, emphasizing the necessity of understanding both additive and non-additive effects when evaluating the ecological risks of surfactant formulations. Furthermore, the observed differential toxicity response in *L. sativum* tissues (roots versus stems) underscores the importance of accounting for species- and tissue-specific sensitivities in environmental impact studies.

Regarding biodegradability, most individual surfactants achieved or were near the 60% mineralization threshold set by the OECD for “readily biodegradable” substances. However, the presence of silica nanoparticles markedly inhibited biodegradation, reducing mineralization rates below this critical threshold. This suggests that nanoparticles may impede microbial processes, either through surfactant adsorption or by forming surfactant/NP complexes that restrict bioavailability. This finding calls for strategic formulation practices to prevent long-term environmental persistence when surfactants are combined with nanoparticles, especially in applications where environmental discharge is probable.

Dermatological assessments using the zein number (ZN) classification revealed that incorporating nanoparticles into surfactant mixtures, particularly those involving FAE-R_12–14_E_11_, could reduce skin irritation potential. This study found that blending nonionic co-surfactants with anionic surfactants, along with nanoparticle inclusion, could further lower skin irritability. This indicates promising pathways for creating safer formulations for products that involve direct human contact. These outcomes are highly dependent on the unique chemical characteristics of the surfactants, and their interactions with nanoparticles require prior study for each case.

In summary, surfactant–nanoparticle formulations incorporating A200 appear to offer a dual effect of reducing acute toxicity and decreasing biodegradability under specific conditions. This research underscores the importance of developing sustainable, eco-friendly surfactant formulations that carefully balance efficacy with minimal environmental and health risks. Future studies should explore these interactions across broader environmental contexts and microbial ecosystems, contributing to more robust regulatory standards and fostering the advancement of greener alternatives in surfactant-based technologies.

## Figures and Tables

**Table 1:** Chemical identification and technical specifications of the surfactants and nanoparticle tested.

Surfactant	Character	INCI Nomenclature	R	E	% Active Matter	HLB	CMC, mg·L^−1^ (±SD)
FAE-R_12–14_E_11_	Nonionic	Laureth-11/Myreth-11	13 ^(a)^	9.9 ^(a)^	97.9 [17]	14.3 [17]	18.5 ± 2.3 [17]
EC-R_12–14_E_10_	Anionic	Laureth-11 Carboxylic Acid	12–14 ^(a)^	10.0 ^(a)^	94.0 [17]	9.5 [17]	70.8 ± 7.2 [17]
EC-R_12–14_E_3_	Anionic	Laureth-4 Carboxylic Acid	12–14 ^(a)^	3 ^(a)^	93.1 [17]	5.5 [17]	33.7 ± 3.1 [17]
EC-R_8_E_5_	Anionic	Capryleth-6 Carboxylic Acid	8 ^(a)^	5 ^(a)^	89.0 [17]	11–14 [17]	97.9 ± 6.5 [17]
Nanoparticle		INCI nomenclature	Dm, nm	S, m^2^·g^−1^	d, g·L^−1^		
A200		Silica	12 [17]	200 ± 25 [17]	50 [17]		

R: alkyl chain length, n-C_i_H_2i+1_-. E: degree of ethoxylation –(OCH_2_CH_2_)_n_O. ^(a)^ Data provided by the supplier.

**Table 2:** Toxicity values (95% CI) of single surfactants.

Surfactant	EC_50_, *V. fischeri* mg·L^−1^	EC_50_, *L. sativum* mg·L^−1^ (Root)	EC_50_, *L. sativum* mg·L^−1^ (Stem)
FAE-R_12–14_E_11_	13.26 ± 3.26	338.17 ± 13.21	97.15 ± 7.14
EC-R_12–14_E_10_	14.18 ± 4.32	32.02 ± 8.90	60.11 ± 6.52
EC-R_12–14_E_3_	3.58 ± 1.28	35.40 ± 4.80	93.25 ± 8.90
EC-R_8_E_5_	32.76 ± 3.60	53.61 ± 7.20	64.77 ± 6.50

**Table 3:** Toxicity values (95% CI) of surfactants and mixtures.

Surfactant A	Surfactant B	NP	EC_50_, *V. fischeri* mg·L^−1^	EC_50_, *L. sativum* mg·L^−1^ (Root)	EC_50_, *L. sativum* mg·L^−1^ (Stem)
FAE-R_12–14_E_11_	EC-R_12–14_E_10_		20.95 ± 3.25	116.41 ± 9.80	66.78 ± 5.60
FAE-R_12–14_E_11_	EC-R_12–14_E_3_		10.78 ± 2.73	86.61 ± 6.21	62.69 ± 8.70
FAE-R_12–14_E_11_	EC-R_8_E_5_		20.64 ± 5.70	39.48 ± 2.75	52.58 ± 3.24
FAE-R_12–14_E_11_		A200	42.21 ± 3.21	788.04 ± 11.22	110.18 ± 6.70
EC-R_12–14_E_10_		A200	49.86 ± 6.95	514.37 ± 15.23	69.49 ± 3.25
EC-R_12–14_E_3_		A200	11.48 ± 4.28	50.69 ± 4.45	58.44 ± 4.45
EC-R_8_E_5_		A200	68.35 ± 5.40	100.79 ± 9.50	91.44 ± 6.42
FAE-R_12–14_E_11_	EC-R_12–14_E_10_	A200	16.86 ± 2.30	430.87 ± 18.47	79.93 ± 6.24
FAE-R_12–14_E_11_	EC-R_12–14_E_3_	A200	15.09 ± 1.25	134.22 ± 10.60	174.52 ± 7.82
FAE-R_12–14_E_11_	EC-R_8_E_5_	A200	48.67 ± 6.70	204.77 ± 9.80	67.33 ± 5.40

**Table 4:** Toxicity values and MTU parameters of surfactant mixtures to bacteria *Vibrio fischeri* (exposure time 30 min) and *Lepidium sativum* (exposure time 72 h).

*Vibrio fischeri*						
Surf. A	Surf. B	TU_A_	TU_B_	TU_mix_	TU_r_	Type of Action
FAE-R_12–14_E_11_	EC-R_12–14_E_10_	0.79	0.74	1.53	2.07	Less than additive (antagonism)
FAE-R_12–14_E_11_	EC-R_12–14_E_3_	0.41	1.51	1.91	1.27	Less than additive (antagonism)
FAE-R_12–14_E_11_	EC-R_8_E_5_	0.78	0.32	1.09	3.47	Concentration addition
*Lepidium sativum* (Root)						
FAE-R_12–14_E_11_	EC-R_12–14_E_10_	0.17	1.82	1.99	1.09	Less than additive (antagonism)
FAE-R_12–14_E_11_	EC-R_12–14_E_3_	0.13	1.22	1.35	1.10	Less than additive (antagonism)
FAE-R_12–14_E_11_	EC-R_8_E_5_	0.06	0.37	0.43	1.16	More than additive (synergism)
*Lepidium sativum* (Stem)						
FAE-R_12–14_E_11_	EC-R_12–14_E_10_	0.34	0.56	0.90	1.62	Concentration addition
FAE-R_12–14_E_11_	EC-R_12–14_E_3_	0.32	0.34	0.66	1.96	More than additive (synergism)
FAE-R_12–14_E_11_	EC-R_8_E_5_	0.27	0.41	0.68	1.67	More than additive (synergism)

**Table 5:** Biodegradation values (95% CI) of single surfactants and binary mixtures (1:1 *w*/*w*).

Surfactant A	Surfactant B	% Mineralization	t_1/2_, h	B, %
FAE-R_12–14_E_11_		61.30 ± 2.60	185.80	2.40
EC-R_12–14_E_10_		69.35 ± 3.21	130.50	10.30
EC-R_12–14_E_3_		57.35 ± 2.80	330.90	1.25
EC-R_8_E_5_		71.20 ± 4.32	118.20	9.40
FAE-R_12–14_E_11_	EC-R_12–14_E_10_	61.49 ± 2.27	564.12	2.71
FAE-_R12–14_E_11_	EC-R_12–14_E_3_	88.10 ± 3.90	265.30	3.35
FAE-R_12–14_E_11_	EC-R_8_E_5_	93.49 ± 3.80	310.20	8.90

**Table 6:** Mineralization values of single surfactants (50 mgThOD·L^−1^) and nanoparticles (250 mg·L^−1^). The theoretical oxygen demand of the solutions tested.

Solution	ThOD, mgO_2_·mg Substance^−1^	% Min
FAE-R_12–14_E_11_ + A200	2.17	54.18
EC-R_12–14_E_10_ + A200	2.20	47.50
EC-R_12–14_E_3_ + A200	1.90	55.28
EC-R_8_E_5_ + A200	1.81	45.25

**Table 7:** Zein number (ZN) (95% CI) of surfactant and mixtures. Concentration of surfactant tested: 0.5% and 1%.

Surfactant A	Surfactant B	NP	ZN 0.5%	ZN 1%
FAE-_R12–14_E_11_			48.91 ± 3.15	55.6 ± 3.20
EC-R_12–14_E_10_			154.69 ± 6.25	201.1 ± 8.40
EC-R_12–14_E_3_			152.92 ± 7.25	183.5 ± 5.60
EC-R_8_E_5_			49.15 ± 3.60	157.9 ± 7.40
FAE-_R12–14_E_11_	EC-R_12–14_E_10_		122.24 ± 5.60	152.8 ± 3.70
FAE-_R12–14_E_11_	EC-R_12–14_E_3_		55.18 ± 4.52	60.7 ± 5.25
FAE-_R12–14_E_11_	EC-R_8_E_5_		40.93 ± 3.20	61.4 ± 2.25
FAE-_R12–14_E_11_		A200	41.06 ± 4.15	73.9 ± 6.75
EC-R_12–14_E_3_		A200	266.08 ± 7.80	345.9 ± 9.45
EC-R_12–14_E_10_		A200	125.59 ± 6.40	213.5 ± 3.25
EC-R_8_E_5_		A200	62.45 ± 4.60	68.7 ± 6.40
FAE-_R12–14_E_11_	EC-R_12–14_E_3_	A200	7.27 ± 1.25	8 ± 1.60
FAE-_R12–14_E_11_	EC-R_12–14_E_10_	A200	34.08 ± 6.35	40,9 ± 3.70
FAE-_R12–14_E_11_	EC-R_8_E_5_	A200	28.81 ± 2.20	46.1 ± 4.25

Skin irritation classification according to the detailed in Göette (1964) [31]: (0–200) non-irritant; (200–400) moderately irritant; (400–500) irritant I; (500–600) irritant II; >600 strongly irritant.

## Data Availability

The data presented in this study are available upon request from the corresponding author.
